# One-Step Plasma Synthesis of Nitrogen-Doped Carbon Nanomesh

**DOI:** 10.3390/nano11040837

**Published:** 2021-03-25

**Authors:** Alenka Vesel, Rok Zaplotnik, Gregor Primc, Luka Pirker, Miran Mozetič

**Affiliations:** 1 Department of Surface Engineering, Jozef Stefan Institute, Jamova Cesta 39, 1000 Ljubljana, Slovenia; rok.zaplotnik@ijs.si (R.Z.); gregor.primc@ijs.si (G.P.); miran.mozetic@ijs.si (M.M.); 2 Department of Condensed Matter Physics, Jozef Stefan Institute, Jamova Cesta 39, 1000 Ljubljana, Slovenia; luka.pirker@ijs.si

**Keywords:** plasma synthesis, one-step procedure, nitrogen doping, carbon nanowalls

## Abstract

A one-step method for plasma synthesis of nitrogen-doped carbon nanomesh is presented. The method involves a molten polymer, which is a source of carbon, and inductively coupled nitrogen plasma, which is a source of highly reactive nitrogen species. The method enables the deposition of the nanocarbon layer at a rate of almost 0.1 µm/s. The deposited nanocarbon is in the form of randomly oriented multilayer graphene nanosheets or nanoflakes with a thickness of several nm and an area of the order of 1000 nm^2^. The concentration of chemically bonded nitrogen on the surface of the film increases with deposition time and saturates at approximately 15 at.%. Initially, the oxygen concentration is up to approximately 10 at.% but decreases with treatment time and finally saturates at approximately 2 at.%. Nitrogen is bonded in various configurations, including graphitic, pyridinic, and pyrrolic nitrogen.

## 1. Introduction

Nitrogen-doped graphene-like nanostructures (hereafter: N-graphene) have attracted significant scientific attention because of their good electronic properties [1,2,3,4] which allows their possible application in electrochemical devices such as fuel cells, super-batteries, and supercapacitors [5,6,7]. As early as 2011, Zhang and Xia provided a feasible explanation for the superior properties of such materials in fuel cells [8]. They provided theoretical backgrounds considering the asymmetry spin density and atomic charge density, making it possible for N-graphene to show high electrocatalytic activities for the oxygen reduction reaction [8]. 

Several techniques for the synthesis of N-graphene have been reported [9,10,11,12,13,14,15]. The most straightforward one is thermal annealing of thin films containing both carbon and nitrogen, usually accompanied by hydrogen. The film is heated to a temperature high enough to enable thermal degradation, in particular de-hydrogenation, and thus the formation of a layer rich in carbon and nitrogen. Such a technique was reported by Kwon et al. [16]. A 20 nm thick film of polypyrrole was deposited onto an activated copper substrate and heated to 900 °C to obtain a graphene-like material with approximately 3 at.% nitrogen, as revealed from X-ray photoelectron spectroscopy (XPS) survey spectra. A somewhat more complicated technique was adopted by Lin et al. [17]. They prepared a composite of graphene oxide and polypyrrole, which was annealed at 800 °C to obtain N-graphene of approximately 2–3 at.% nitrogen and a porous structure with micro-and mesopores of high catalytic activity. 

Porous structures with dense N-graphene can also be synthesized at lower temperatures. An established technique for synthesizing nanomaterials is hydrothermal treatment. Sun et al. [18] used graphene oxide and urea to synthesize N-graphene of a large surface and with an excellent capacitive performance at 180 °C. The nitrogen content was as large as 11 at.% using the hydrothermal technique. 

An alternative to such classical techniques is the application of highly non-equilibrium gaseous plasma. Jeong et al. [19] synthesized high-performance ultracapacitors by performing the reduction of graphene oxide in hydrogen plasma, followed by further treatment in the nitrogen plasma. The final treatment step was annealing at 300 °C to remove residual functional groups. A similar technique with graphene oxide as the raw material and application of a gas mixture containing hydrogen and nitrogen or ammonia was used by Zhang et al. [20] and Kumar et al. [21]. An overview of plasma-based techniques for the synthesis of N-graphene was published recently in [22]. 

The methods mentioned above enable the synthesis of high-quality N-graphene, but the applicability is limited because of rather long treatment times. Mass application of such materials is possible only when using a technique that enables rapid deposition, preferable in a continuous mode. Traditional plasma techniques rely on introducing a carbon- and a nitrogen-containing gas into the plasma reactor simultaneously [14]. The carbon-containing gas could be methane and any other light hydrocarbon or a monomer containing nitrogen. The source of nitrogen could be N_2_ or NH_3_. The precursors are partially decomposed upon plasma conditions, and the radicals condense on a surface facing plasma to form any coating from thin films of hydrogenated carbon [23,24] to highly-oriented carbon nanowalls [25,26]. The growth rate, however, is limited by the scavenger effect: the dominant product is gaseous hydrogen cyanide [27]. The growth rate may be increased by increasing the pressure in the plasma reactor; however, a rather large density of precursors in gaseous plasma causes the formation of dust particles in the gas phase, which is often detrimental to the quality of the deposits [28].

The upper limitations may be avoided by using an alternative source of carbon in the plasma reactor. Graphite as a raw material seems to be a promising source for synthesizing N-graphene nanostructures; however, it will not be etched at sufficient rates upon treatment with nitrogen plasma at a reasonable temperature to assure for rapid deposition of thin films. A substitute for graphite could be aromatic polymers. Such polymers decompose at elevated temperatures forming conjugated aromatic rings [29]. The aromatic rings interact with reactive nitrogen species and enter the plasma where they radicalize and represent building blocks for N-doped graphene nanomesh. 

In the present paper, we introduce a novel direct one-step method for fast synthesis of N-doped carbon nanomesh using nitrogen plasma as a nitrogen source and a polymer material as a source of carbon precursor.

## 2. Materials and Methods

### 2.1. Plasma Synthesis

**Figure 1:**
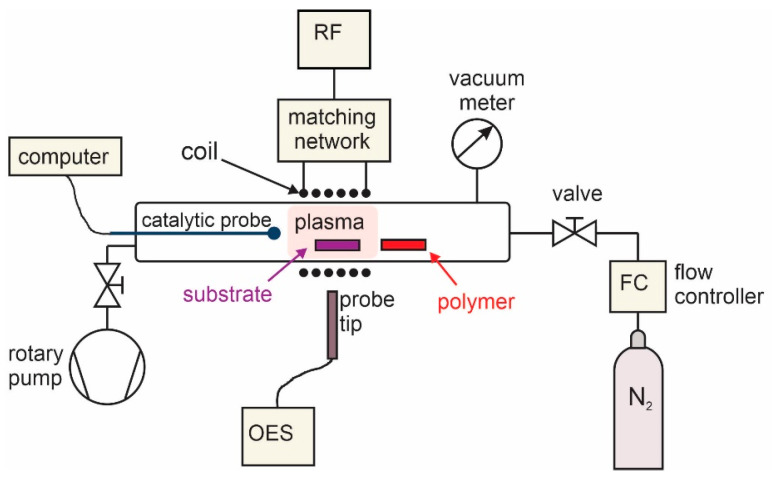
Experimental set-up.

The experimental set-up used for the deposition of N-doped carbon nanomesh is shown schematically in Figure 1. The plasma reactor employs an inductively coupled radiofrequency (RF) discharge to sustain the rather dense and uniform plasma in nitrogen. A coil with six turns was mounted on the discharge tube, which was pumped with a rotary pump with a nominal pumping speed of 80 m^3^/h. The base pressure was approximately 1 Pa. Titanium foil was used as a substrate for the deposition of N-doped carbon nanomesh. Ti substrates of dimensions 8 mm × 8 mm × 0.1 mm were placed into a glass discharge tube together with a sample of polymer polyethylene terephthalate (PET), as shown in Figure 1. The dimensions of the PET foil were 20 mm × 20 mm × 0.25 mm. N_2_ gas of commercial purity 99.999% was used as a nitrogen source, and the PET polymer was a source of carbon. Nitrogen pressure in the discharge tube was set to 15 Pa. The plasma was ignited and sustained at the forward power of the RF generator of 500 W. At these conditions, nitrogen plasma was sustained in the H-mode, where the absorbed power was high. Ti substrate was heated in the plasma because of exothermic heterogeneous surface reactions and reached a temperature of approximately 800 °C after several seconds. The deposition time was varied from 10 to 120 s.

The N-atom density in nitrogen plasma was measured with a catalytic probe, and it was approximately 0.4 × 10^20^ m^−3^. Plasma was also characterized by optical emission spectroscopy (OES) using an AvaSpec-3648 Fiber Optic Spectrometer (Avantes, Apeldoorn, The Netherlands). The spectrometer resolution was 0.5 nm in the range of wavelengths between 200 to 1100 nm. The integration time was 1 ms.

### 2.2. Characterization of the Samples

#### 2.2.1. X-ray Photoelectron Spectroscopy (XPS)

XPS characterization was performed by using an XPS (TFA XPS Physical Electronics, Münich, Germany). The samples were excited with monochromatic Al Kα_1,2_ radiation at 1486.6 eV over an area with a diameter of 400 µm. Photoelectrons were detected with a hemispherical analyzer positioned at an angle of 45° with respect to the normal of the sample surface. Survey spectra were measured to determine the nitrogen content in the samples. High-resolution spectra of C1s and N1s were also measured. The C1s and N1s spectra were measured at a pass-energy of 23.5 eV using an energy step of 0.1 eV. Auger CKLL spectrum (1190–1250 eV) was also measured with a pass-energy of 117.4 eV using an energy step of 0.25 eV. The measured spectra were analyzed using MultiPak v8.1c software (Ulvac-Phi Inc., Kanagawa, Japan, 2006) from Physical Electronics, which was supplied with the spectrometer. Shirley background subtraction was used. No flood gun for charge neutralization was needed. The binding energy was corrected by taking sp^2^ carbon for a reference at 284.4 eV. The sp^2^ carbon was fitted with an asymmetric function determined on highly-oriented pyrolytic graphite (HOPG) reference. 

#### 2.2.2. Time-of-Flight Secondary Ion Mass Spectrometry (ToF-SIMS)

Time-of-flight secondary ion mass spectrometry (ToF-SIMS) analyses of the sample deposited at 60 s of plasma exposure were performed using a ToF-SIMS 5 instrument (ION-TOF, Münster, Germany). The instrument was equipped with a bismuth liquid metal ion gun with a kinetic energy of 30 keV. The analyses were performed in an ultra-high vacuum of approximately 10^−7^ Pa. The SIMS spectra were measured by scanning an ion beam of Bi^3+^ clusters. The diameter of the beam was 1 μm, and it was scanned over an area of 500 × 500 μm^2^. The negative secondary ion mass spectra were measured to obtain the characteristic mass fragments. Imaging of the surface was performed on an area of 20 × 20 μm^2^. Depth profiling was also performed using Cs^+^ ions with an energy of 2 keV to reveal nitrogen distribution in the deposit.

#### 2.2.3. Raman Spectroscopy

Raman spectroscopy was performed using a WITec Alpha 300 RS scanning confocal Raman microscope (WITec GmbH, Lise-Meitner, Germany) in backscattered geometry with a polarized Nd:YAG laser operating at the 532 nm wavelength and a He:Ne laser operating at 633 nm wavelength. The laser beam was focused through a 100×/0.9 microscope objective. The power of the laser was approximately 1 mW.

#### 2.2.4. Scanning Electron Microscopy (SEM) and Transmission Electron Microscopy (TEM) 

Scanning electron microscopy (SEM) micrographs were acquired using a Schottky field-emission electron microscope Jeol JSM-7600F (Jeol Ltd., Tokyo, Japan). It is a semi in-lens SEM with the adoption of a high-power optics irradiation system that delivers high-resolution, high-speed, and high-accuracy element analysis. SEM analyses were performed without any coating of the samples because they are conductive enough to prevent any charge accumulation during the imaging. A sample was placed onto a stainless steel holder and fixed with adhesive carbon tape. The primary electron beam was rastered on the surface of an area of approximately 6 µm^2,^ and the image was obtained from secondary electrons. The electron kinetic energy was 5 keV and the sample’s working distance (from the pole piece) was 4.5 mm.

Transmission electron microscopy (TEM) images were acquired using a transmission electron microscope JEM-2100 (Jeol Ltd., Tokyo, Japan). The sample was scratched directly to the lacey carbon-supported copper grid. Detailed structural investigations of the sample were performed using a conventional 200 kV TEM equipped with a LaB_6_ electron source.

## 3. Results and Discussion

### 3.1. Synthesis Mechanism of N-Doped Carbon Nanomesh

Nitrogen plasma at our experimental conditions was rich with nitrogen atoms, ions, as well as with N_2_ molecules in various rotational and vibrational states. Because of the large density of reactive species and a high vibrational temperature, which is typical for N_2_ plasma [30], it caused heating and melting of the polymer. The reactive nitrogen species interacted chemically with the molten polymer, forming various molecules containing carbon, nitrogen, and hydrogen. The molecules desorbed from the surface of the molten polymer and entered the gas phase, where they were partially decomposed and ionized upon plasma conditions. Nitrogen-containing carbon fragments condensed on the substrate surface, forming a deposit of carbon nanomesh rich with nitrogen. Such condensation is typical for plasma-enhanced chemical vapor deposition (PECVD) and is explained by a high sticking coefficient of plasma radicals on a surface of a material facing the plasma. A moderately high substrate temperature prevented hydrogenation of the deposited film. The substrate temperature was approximately 800 °C in our case. Because of the low thermal capacity of the titanium substrate and high power absorption in plasma in the H-mode, both the substrate and the polymer sample were heated to elevated temperatures within a few seconds after turning on the discharge. 

**Figure 2:**
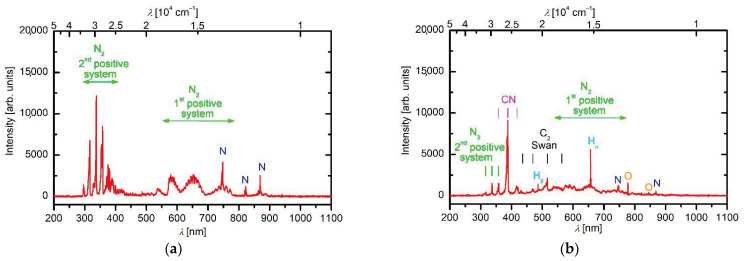
Optical emission spectroscopy (OES) spectrum of nitrogen plasma: (**a**) without any sample, and (**b**) during carbon nanomesh deposition.

Reactive plasma species during deposition of N-doped carbon nanomesh were monitored by OES. Figure 2 shows a comparison of the OES spectra of N_2_ plasma without and with the polymer sample. For plasma without the sample (Figure 2a), nitrogen atomic lines, as well as nitrogen molecular bands arising from the 1st and 2nd N_2_ positive systems, were observed. These transitions arise from B^3^Π_g_ and C^3^Π_u_ states and terminate in A^3^∑_u_^+^ and B^3^Π_g_ states, respectively. The potential energy of the basic vibration state for the B^3^ Π_g_ and C^3^ Π_u_ levels is at approximately 7.4 and 11.1 eV, respectively. The nitrogen molecules are found at a high vibrational temperature in low-pressure plasmas [31], so the spectrum in Figure 2a contains numerous features corresponding to transitions from various vibrational states to a lower electronic level of numerous vibrational states. Furthermore, the rotational temperature is not negligible in inductively coupled plasma in the H-mode, which adds to the complexity of the spectrum in Figure 2a. Nevertheless, the nitrogen-plasma spectrum qualitatively indicates a good excitation of the states of rather high potential energy. Such states are chemically reactive. Furthermore, clearly distinguished N-atomic lines in the near infrared (IR) part of the spectrum also indicate a significant dissociation fraction. The OES spectrum during the carbon nanomesh deposition is shown in Figure 2b. Although N-atomic lines and the N_2_ positive systems are still observed, the spectrum is now dominated by the CN band, C_2_ Swan band, as well as with oxygen and hydrogen atomic lines (H_α_, H_β_). These spectral features indicate the partial degradation of the molecules formed on the surface of the molten polymer upon plasma exposure. The OES, unfortunately, does not allow for observing larger molecules. Still, it gives an insight into the plasma chemistry upon the interaction of reactive nitrogen species with the molten polymer. The intensive band arising from CN is expected because HCN is the simplest molecule that is formed as a consequence of the etching of the organic material with nitrogen plasma. Furthermore, the triple bond C≡N is among the strongest chemical bonds at 887 kJ/mol (9.2 eV), thus it is unlikely to break the bond even upon plasma conditions. More interesting is the absence of the atomic carbon line (which should appear at 248 nm) [32] and a rather intensive Swan band, which is a consequence of radiative relaxation of C_2_ dimers. The absence of the C-line indicates incomplete radicalization of the nitrated aromatic hydrocarbons, which are likely to be formed upon nitrogen interaction with hydrocarbons at high temperatures [33]. The Swan band indicates that a fraction of the molecules formed upon etching of the molten polymers with nitrogen plasma is decomposed to the C_2_ dimers. Therefore, the plasma chemistry enables the partial decomposition of etching products in the gas phase and condensation of such products on the substrate surface. The substrate’s high temperature assures at least partial dehydrogenization and thus the growth of a film consisting of N-doped graphene.

### 3.2. Surface Characterization of N-Doped Carbon Nanomesh

**Figure 3:**
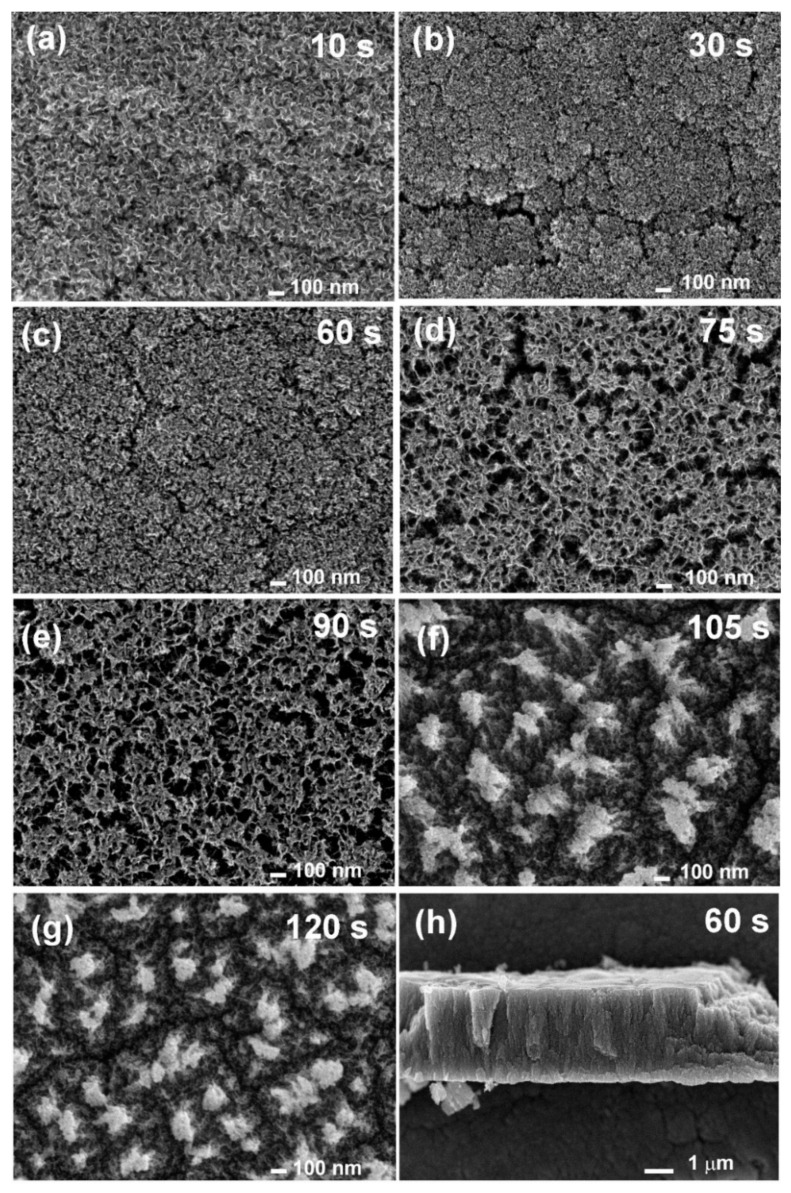
(**a**–**g**) Scanning electron microscopy (SEM) images of N-doped carbon nanomesh deposited at various deposition times, and (**h**) cross-section of the deposited formed at 60 s of treatment.

The N-doped carbon nanomesh was deposited at various treatment times between 10 and 120 s. Figure 3 shows SEM images of N-doped carbon nanomesh samples. We can see already that 10 s of exposure to N_2_ plasma was enough to form a dense mesh of nanoflakes (Figure 3a). The morphology of nanoflakes did not change much till approximately 60 s of plasma deposition time (Figure 3b,c). At longer deposition times (Figure 3d,e), changes in surface morphology were observed. Figure 3d,e reveal something resembling the clustering of the deposits leading to the formation of gaps of a width of approximately 100 nm. Clustering is even more pronounced after long treatment (Figure 3f,g), where some agglomerates are observed. Such agglomerates may be detrimental to the porosity of the deposited material; therefore, it is regarded as an unwanted effect. The agglomeration may appear because of increased surface temperature. Namely, the discharge parameters were independent of the treatment time. The film consisting of N-doped carbon nanomesh has poor thermal conductivity because of the high porosity; therefore, the surface temperature may increase with increasing thickness and thus treatment time. The thickness of the deposited film of N-doped carbon nanomesh was measured after several treatment times. At 20 s of treatment, it was just below 1 μm, and it increased to approximately 3.5 µm for a minute of treatment time, as revealed from Figure 3h. The thickness of the deposit was about 5 μm for the longest deposition time of 120 s. Poor increase of the film thickness between 60 and 120 s of treatment may be a consequence of the agglomeration. 

**Figure 4:**
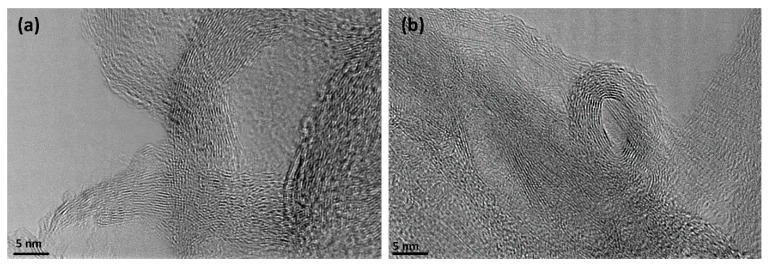
Transmission electron microscopy (TEM) image of N-doped graphene showing graphene layers: (**a**) 60 s of treatment, and (**b**) 105 s of treatment.

Additionally to SEM characterization, TEM analyses were also performed to confirm the structure of the carbon nanomesh. For convenience, an example of a TEM image is shown in Figure 4 for the samples treated for 60 (Figure 4a) and 105 s (Figure 4b). Graphene layers can be observed in Figure 4 for both treatments. The distance between the neighboring layers of graphene was estimated to be approximately 0.36 nm, which is in agreement with other reports [34,35]. Despite the fact that the SEM image of the sample in Figure 3f already shows signs of agglomeration, the graphene-like structure was still preserved.

**Figure 5:**
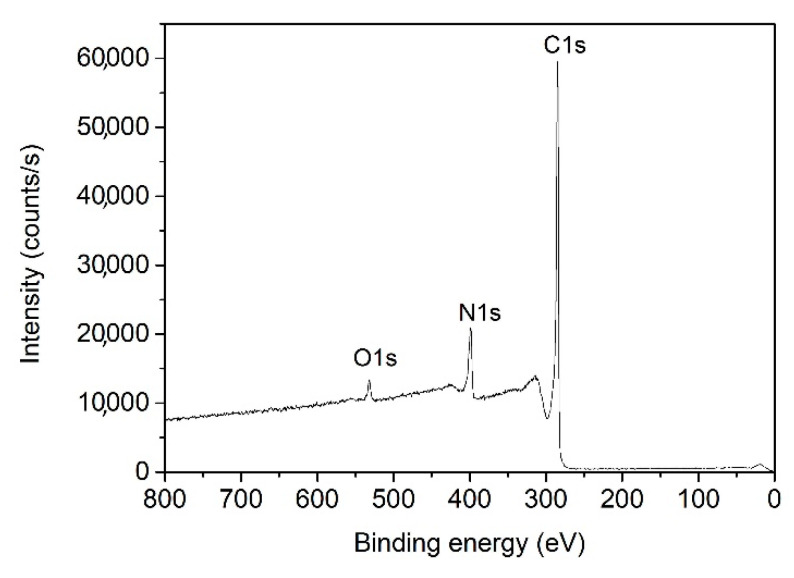
X-ray photoelectron spectroscopy (XPS) survey spectrum of N-doped carbon nanomesh sample with deposition time of 105 s.

Samples were further characterized by XPS to determine their chemical composition. Figure 5 shows an example of the XPS survey spectrum of the sample deposited at 105 s treatment time, showing its surface elemental composition. In addition to carbon, nitrogen is clearly visible as well as some oxygen. The detailed surface composition of the N-doped carbon nanomesh deposited at various treatment times is shown in Table 1. Nitrogen concentration in the samples increased with plasma treatment time. At low deposition times, nitrogen concentration was approximately 1 at.%, and for the longest deposition time, it increased to almost 15 at.%. For oxygen concentration, the opposite trend was observed; i.e., it decreased with increasing deposition time. The results in Table 1 thus confirm the successful enrichment of carbon nanomesh with nitrogen with increasing treatment time, whereas the oxygen remained within the tolerable concentration. 

A significant concentration of oxygen on the surface of N-doped graphene was observed by all authors, including [11,19,36,37,38], and was explained by a higher susceptibility of the N-doped graphene to functionalization with oxygen [39]. A feasible explanation for our observation, i.e., decreasing O-concentration and increasing N-concentration, could be in thermal degradation of the PET material. Thermal cleavage of the ester bonds in the PET backbone results in the formation of water vapor and carbon dioxide, leaving the surface rich in conjugated aromatic rings as shown by Holland et al. [29] and Turnbull et al. [40]. The initial stage of plasma treatment, therefore, involves the desorption of gases rich in oxygen (H_2_O and CO_2_) which radicalize to highly oxidizing OH and O radicals upon plasma conditions. The initial stage of deposition is therefore governed by excessive oxidizing radicals, which cause significant oxidation of the carbon nanomesh. As the plasma treatment prolongs, the supply of oxidizing radicals is suppressed, so the deposits assume the composition of only 2 at.% of oxygen after about a minute of plasma treatment. Simultaneously, the concentration of nitrogen in the deposits increased because they occupy the binding sites at the edge defects of the graphene structure. Another explanation for decreasing O-concentration with treatment time may also be thermal effects. Kundu et al. [41] have investigated thermal stability of oxygen functional groups on carbon nanostructures and found decomposition of carboxylic groups already at 300 °C. The authors also found that oxygen concentration decreased from 10.7 to 4.3 at.% when the temperature was increased from 300 to 720 °C.

**Figure 6:**
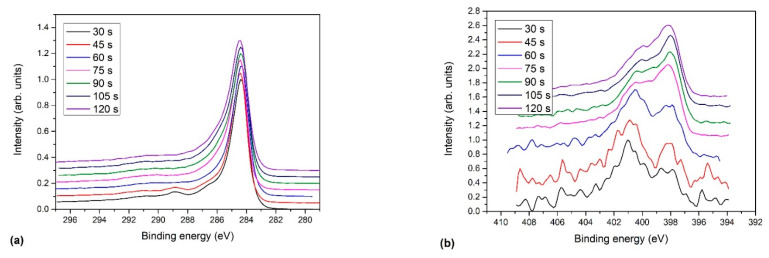
Evolution of: (**a**) high-resolution C1s, and (**b**) N1s spectra of N-doped carbon nanomesh versus deposition time.

Figure 6 shows the evolution of high-resolution carbon C1s and nitrogen N1s spectra with deposition time. Figure 6a reveals peaks at approximately 289 eV for the lowest treatment times (30 and 45 s), which are not pronounced at longer treatment times. The peak at this binding energy is characteristic of highly oxidized carbon, for example, the O=C-O group. The rather large concentration of oxygen at these short treatment times favors the formation of such a carbon-oxygen functional group with high oxygen content. The absence of the peak at longer treatment times could be explained either by the shortage of oxygen in the gas phase or thermal degradation, as explained in [41].

While the carbon C1s spectra in Figure 6a were rather similar, large differences were observed for the nitrogen N1s spectra shown in Figure 6b, indicating that the ratio between various nitrogen configurations was changing with treatment time. We can observe two trends. Up to 60 s of treatment, the N1s peaks at high-binding energies were dominant, whereas, at treatment times above 75 s, the peaks at lower binding energy prevailed. It has been reported that nitrogen in graphene usually appears in three typical configurations: pyridinic, pyrrolic, and quaternary (graphitic) nitrogen [12,39,42,43]. 

**Figure 7:**
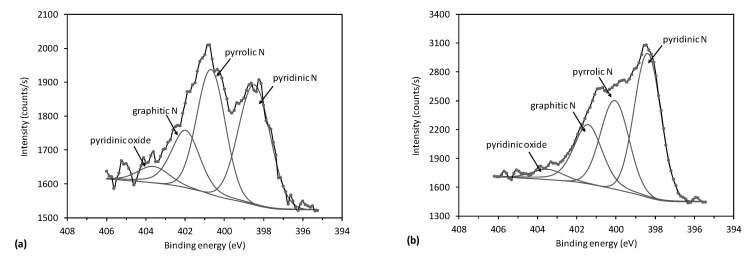
High-resolution nitrogen spectra N1s for N-doped carbon sample treated for (**a**) 60 s, and (**b**) 90 s.

In Figure 7a,b are examples of N1s spectra with subcomponents showing nitrogen configurations for 60 and 90 s of treatment, respectively. The spectra were fitted with four peaks, positioned at 398.6, 400.3, 401.6, and 403.5 eV, which were assigned to pyridinic nitrogen, pyrrolic nitrogen, graphitic nitrogen, and oxidized nitrogen groups, respectively. While graphitic nitrogen concentration did not change noticeably with deposition time, remarkable variation was observed for pyrrolic and pyridinic nitrogen with treatment time. At low deposition times when nitrogen concentration was low, pyrrolic nitrogen prevailed (Figure 7a). In contrast, at high deposition times, where a high nitrogen concentration in the sample was measured, pyridinic nitrogen became dominant (Figure 7b). Pyrrolic nitrogen is bonded to a hydrogen atom, while pyridinic is free from hydrogen. The evolution of both configurations, as observed from XPS results, is sound with the reports of other authors mentioned in the Introduction regarding the thermal effects: prolonged treatment times cause de-hydrogenation because of the higher surface temperature. 

**Figure 8:**
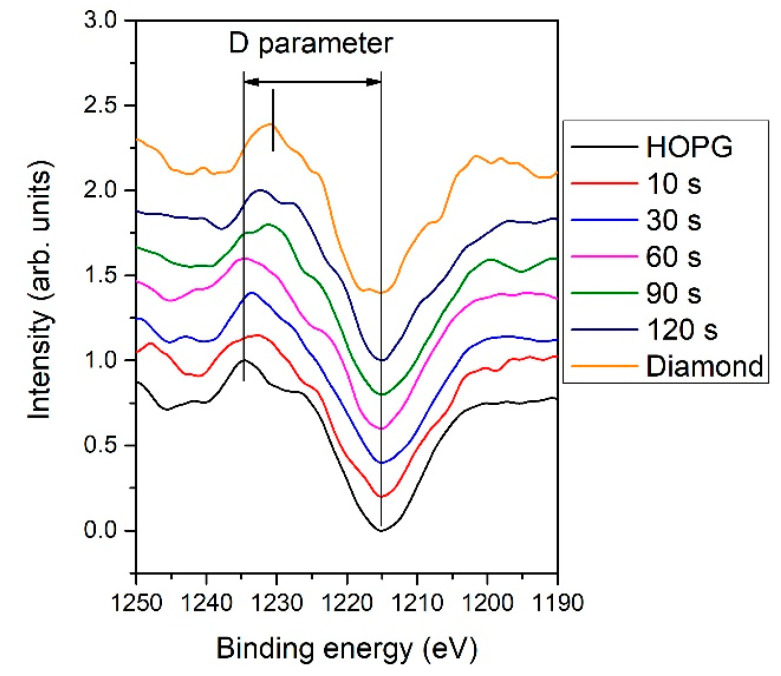
The first derivative of Auger CKLL spectra of N-doped carbon nanomesh deposited at various times and of highly-oriented pyrolytic graphite (HOPG) and diamond reference samples.

In addition to XPS high-resolution spectra, the first-derivatives of Auger CKLL spectra acquired with the XPS instrument were also examined and are shown in Figure 8. The distance between the minimum and maximum is known as the “D-parameter”. It was reported that the D-parameter depends on the ratio between sp^2^ and sp^3^ carbon configurations, being larger for sp^2^ than for sp^3^ [44,45]. According to the literature, the reported D-parameter was D ≈ 21 and 14 eV for graphite and diamond, respectively [44,45]. However, we should also note that this approach may be uncertain because the determination of the exact position of the minimum and maximum in the Auger CKLL spectrum is not completely reliable. Nevertheless, in our case, we used reference materials such as HOPG and a diamond powder and obtained values of D ≈ 19.4 and 13.7 eV, respectively. For N-doped carbon nanomesh, the D-parameter for short deposition times up to 60 s was similar to HOPG, i.e., D ≈ 18.8, whereas for longer deposition times, it decreased to approximately D ≈ 16.8. This observation indicates a decrease of sp^2^ content with increasing deposition times. By considering SEM images of Figure 3, one may conclude that the agglomerates observed in Figure 3f,g are rich in sp^3^.

**Figure 9:**
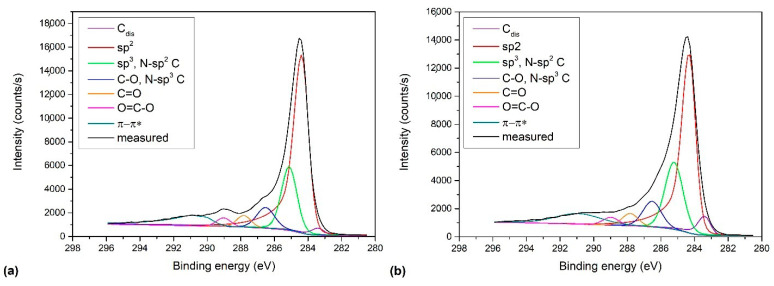
Selected C1s spectra of as-deposited N-doped carbon nanomesh samples. The deposition times were: (**a**) 30 s, and (**b**) 120 s.

**Figure 10:**
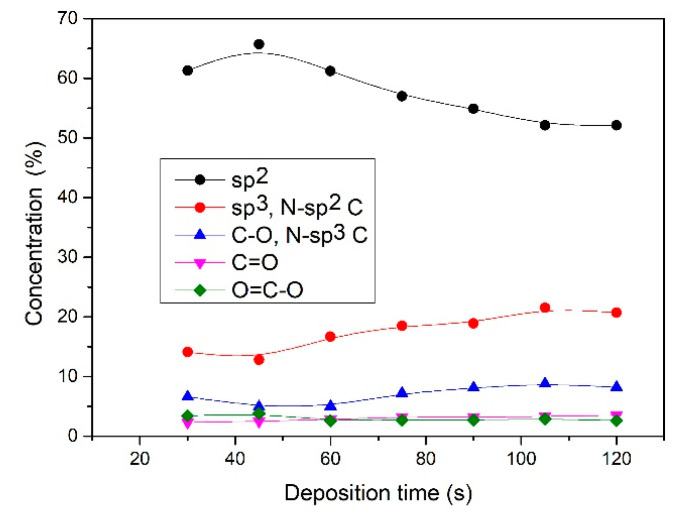
Evolution of chemical structure with deposition time.

To further investigate the nature of carbon configuration, an attempt was made to fit the carbon C1s spectra shown in Figure 6a. Because the asymmetric shape line is characteristic of graphene [43,46,47], the spectra were fitted with the asymmetric peak previously acquired using the HOPG reference. It is known that the main sp^2^ carbon peak for graphene domains is positioned at 284.4 eV [48,49]. The spectrum of graphene also displays several peaks caused by energy losses because of π-π* excitation, which are positioned on the high binding energy side and extend several eV from approximately 289 eV onward [50]. In our case, these π-π* features were fitted with one broad peak. Defects induced to the graphene structure during doping cause a change in the line shape on both high- and low-binding energy sides. As commonly adopted in literature, sp^3^ C is positioned up to about 1 eV at higher binding energy than sp^2^. It was also reported that at the same time, another peak C_dis_ corresponding to the introduction of defects in sp^2^ carbon could be observed at lower binding energies of approximately 284.0 eV [11,47,50,51]. In the range between 285 and 289 eV, peaks arising from carbon-nitrogen and carbon-oxygen bonds appear and partially overlap. For N-doped samples, peaks corresponding to sp^2^ C=N and sp^3^ C-N should appear at approximately 285.5–286.3 eV and 286.9–288.3 eV, respectively [13,48,52,53,54]. By considering all these facts, the C1s peak was fitted with six subpeaks, which correspond to C_dis_ (peak 1), sp^2^ C (peak 2), sp^3^ C and sp^2^ C-N (peak 3), C-O, and sp^3^ C-N (peak 4), C=O and 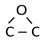 (peak 5), O=C-O (peak 6), and π-π* excitation. Two examples are shown in Figure 9. Figure 9a shows an example of the C1s peak for short deposition times, whereas Figure 9b for long deposition times. The results for all samples obtained from fitting as shown in Figure 9 are summarized in Figure 10. Figure 10 shows the evolution of functional groups with deposition time. Here we should also note that overlapping of the O=C-O peak at ~289 eV with the π-π* peak brings some uncertainties in determining the corresponding concentrations. In Figure 10, we can observe a decreasing concentration of sp^2^ carbon with deposition time, which is in agreement with the results of the D-parameter shown in Figure 8. We can also observe the increasing intensity of peaks related to sp^3^ and nitrogen content. The detailed analyses of the C1s peak, therefore, support the conclusion brought on the basis of the Auger CKLL spectra. Here, the overlapping of the oxygen and nitrogen-containing functional groups should be stressed again. For example, the functionality marked as “C-O and sp^3^ C-N” is likely to be almost free from oxygen at high deposition times.

**Figure 11:**
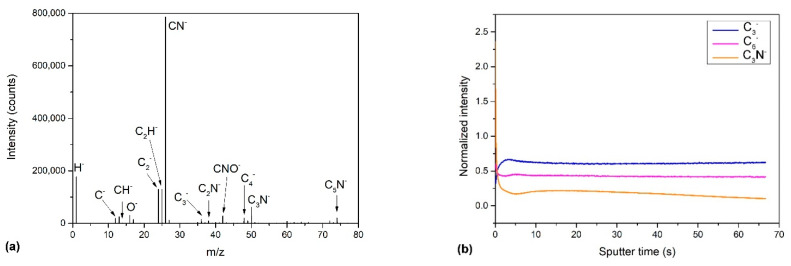
(**a**) Secondary ion mass spectrometry (SIMS) negative ion spectrum of the N-doped carbon nanomesh sample (deposition time 60 s) and (**b**) SIMS depth profile of this sample.

ToF-SIMS measurements support the XPS results. Figure 11a shows the SIMS spectrum of negative ions of the selected sample treated for 60 s. This sample was chosen because of the highest content of the pyrrolic nitrogen configuration and because of the uniform deposit with a lack of any agglomeration, as revealed by SEM (Figure 3c). Figure 11a reveals characteristic peaks belonging to carbon-containing fragments from graphene (C^−^, C_2_^−^, C_3_^−^, C_4_^−^) as well as fragments containing nitrogen (CN^−^, C_2_N^−^, C_3_N^−^, C_5_N^−^) and oxygen (CNO^−^). Depth profiling was also performed to investigate the distribution of nitrogen within the sample. The depth profiles are shown in Figure 11b. The variation of characteristic fragments is shown versus sputtering time. The sputtering rate was roughly estimated at 0.2 nm/s. We can see that nitrogen is quite uniformly distributed in the sample because the signal is rather stable, and only a minor decrease in the intensity of the C_3_N^−^ is observed. However, such a decrease can also be an artifact because of the matrix effects typical for the SIMS technique.

**Figure 12:**
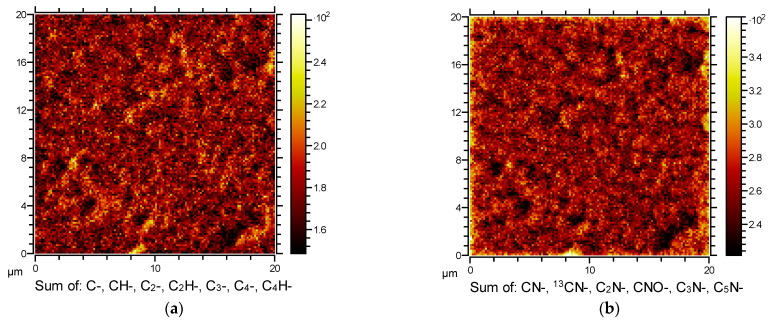
2D SIMS image of N-doped carbon nanomesh surface: (**a**) distribution of carbon fragments, and (**b**) distribution of nitrogen-containing fragments. The deposition time was 60 s.

Furthermore, carbon is known as a material with a very low sputtering yield. In addition, surface topography has a significant effect on the sputtering yield because of various alignments of nanowalls and, thus, different incidence angles of impinging ions [55]. It can also be expected that the sputtering yield may change during the sputtering of the sample. All these effects, therefore, cause difficulties in the interpretation of the SIMS results. Some variation of the signal observed just at the surface after starting the etching is probably because of surface-induced effects. Additionally, we show in Figure 12 two dimensional SIMS mapping of the surface of the N-doped carbon nanomesh. Figure 12a illustrates the distribution of the carbon fragments (sum of C^−^, CH^−^, C_2_^−^, C_2_H^−^, C_3_^−^, C_4_^−^, C_4_H^−^), whereas Figure 12b shows the distribution of the nitrogen-containing fragments (sum of CN^−^, C_2_N^−^, CNO^−^, C_3_N^−^, C_5_N^−^). This is opposite to the results of Luo et al., who found non-uniform nitrogen distribution for the sample synthesized by CVD in NH_3_/He combined with C_2_H_4_/H_2_ gas [56], the surface in our case is very homogenous indicating uniform nitrogen distribution.

**Figure 13:**
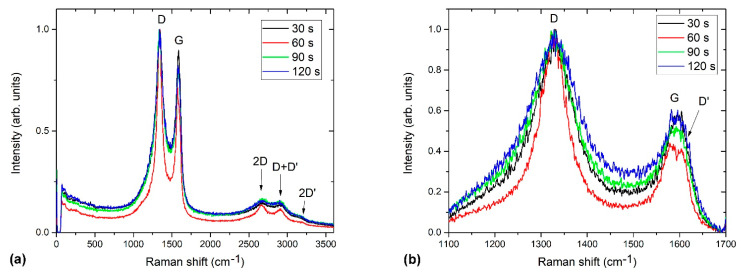
(**a**) Comparison of Raman spectra of N-doped carbon nanomesh samples, (**b**) a detail showing a D’ band.

**Figure 14:**
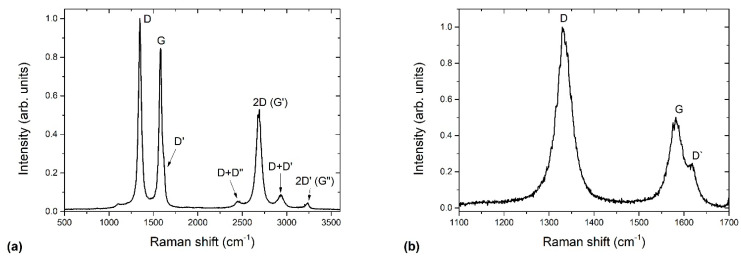
(**a**) Raman spectra of a reference undoped carbon nanomesh, (**b**) a detail showing a D’ band.

Raman spectra of selected samples are shown in Figure 13a. The main D and G bands are ploted in more detail in Figure 13b. As a reference, we also show in Figure 14 an example of pure undoped carbon nanomesh deposited at similar plasma conditions. The Raman spectrum of the reference undoped carbon nanomesh (Figure 14a) shows a typical G band, characteristic for crystalline graphite (sp^2^ carbon domains), and a D band, characteristic for disordered structure, i.e., graphene edges. The G band peak is accompanied by a shoulder corresponding to D` band, as shown in detail in Figure 14b. The D and D` bands are associated with the presence of the edges of graphene nanowalls and defects and thus also with more nanocrystalline structures [57]. In Figure 14a, we can also observe their combination modes and second-order bands: D + D’’, 2D, D + D’, and 2D’.

The Raman spectra of N-doped carbon nanomesh (Figure 13) considerably differ from Figure 14. In Figure 13 we can observe a significant broadening of all peaks. Furthermore, the intensity of the 2D band is much lower for the case of N-doped than pristine materials. The D’ band cannot be distinguished anymore from the G band, except for the sample treated for 60 s (Figure 13b). The spectrum in Figure 13 is similar to those observed by Manojkumar et al. [58] for the case of nitrogen implanted vertical graphene nanowalls. 

The broadening of Raman spectra can be explained by amorphization, as reported by Lucchese et al. [59]. Lucchese [59] exposed graphene to various doses of Ar^+^ ions and investigated the evolution of Raman spectra. They found that the intensity of the disordered D band increased with an increasing dose; however, at a dose of 10^14^ cm^−2^, the intensity of the D band decreased and significantly broadened, which was explained by amorphization and partial sputtering of the sample and thus more structural disorder. From Figure 13 we can thus conclude that our N-doped carbon nanomesh has a higher degree of amorphization than undoped carbon nanomesh.

In Figure 13 we can also notice that the curve for the sample treated for 60 s is the narrowest. This can be explained by the time evolution of the carbon nanomesh growth. As revealed by SEM, after the initial formation of carbon nanomesh, the most uniform deposit is formed at 60 s of treatment before agglomerates begin to appear with the further increase of treatment time

## 4. Conclusions

A method for direct plasma synthesis of N-doped carbon nanomesh was presented. The N-doped carbon nanomesh was deposited on Ti substrates by a PECVD method using nitrogen plasma and molten polymer as a carbon source. Nitrogen radicals reacted with the polymer, causing its melting and desorption of nitrogen-containing carbon fragments on the Ti substrate. This effect enabled the formation of a layer of N-doped carbon nanomesh on the substrate. The deposition rate depended on the evolution of the film, but on average, it was approximately 3 μm/min. The nitrogen concentration in the deposit increased with deposition time from approximately 1 to almost 15 at.%. However, as shown by various characterization techniques including SEM, TEM, Raman, and XPS, the quality of the deposited N-doped carbon nanomesh was better at a deposition time of 60 s as long as the merit is the uniform surface morphology and high sp^2^/sp^3^ ratio. If the nitrogen content is the merit, longer deposition time is better; however, one must bear in mind that the sp^2^/sp^3^ ratio is lower. With longer treatment time the predominant nitrogen configuration also changed from pyrrolic to pyridinic and some agglomerates were formed. The concentration of graphitic nitrogen was more or less independent of treatment time.

## Figures and Tables

**Table 1:** Surface composition of as-deposited N-doped carbon nanomesh versus deposition time.

Time (s)	C (at.%)	N (at.%)	O (at.%)	N/C (%)	O/C (%)
10	91.4	0.8	7.9	0.8	8.6
20	90.6	2.2	7.2	2.4	7.9
30	90.2	1.3	8.5	1.5	9.4
45	91.6	1.4	7.1	1.5	7.7
60	93.8	3.0	3.2	3.2	3.4
75	89.9	8.3	1.8	9.3	2.0
90	88.2	10.2	1.6	11.6	1.8
105	85.6	12.7	1.7	14.8	2.0
120	85.7	12.5	1.7	14.6	2.0

## Data Availability

Data is contained within the article.
